# Quantitative evaluation of embedding resins for volume electron microscopy

**DOI:** 10.3389/fnins.2024.1286991

**Published:** 2024-02-09

**Authors:** Lennart Tegethoff, Kevin L. Briggman

**Affiliations:** Max Planck Institute for Neurobiology of Behavior - caesar, Bonn, Germany

**Keywords:** epoxy resin, volume electron microscopy (vEM), hardness, cutting forces, tissue embedding

## Abstract

Optimal epoxy resin embedding is crucial for obtaining consistent serial sections from large tissue samples, especially for block faces spanning >1 mm^2^. We report a method to quantify non-uniformity in resin curing using block hardness measurements from block faces. We identify conditions that lead to non-uniform curing as well as a procedure to monitor the hardness of blocks for a wide range of common epoxy resins used for volume electron microscopy. We also assess cutting repeatability and uniformity by quantifying the transverse and sectional cutting forces during ultrathin sectioning using a sample-mounted force sensor. Our findings indicate that screening and optimizing resin formulations is required to achieve the best repeatability in terms of section thickness. Finally, we explore the encapsulation of irregularly shaped tissue samples in a gelatin matrix prior to epoxy resin embedding to yield more uniform sections.

## Introduction

Several methods are now available for the automated collection of large volumetric electron microscopy (EM) datasets (Briggman and Bock, 2012; Peddie et al., 2022). All of these methods rely on the embedding of stained biological tissue in various resins to stabilize the tissue before thin sectioning or ion ablation. The choice of the optimal embedding resin has been, in our hands, a matter of trial and error and has lacked metrics on the sectioning properties of resins to help guide the decision. This is perhaps why the resins utilized in recent connectomic reconstructions have so widely varied including the use of Spurr’s resin (Karimi et al., 2020; Loomba et al., 2022), Durcupan (Calì et al., 2018), and Epon 812 replacements such as EMbed 812 (Kasthuri et al., 2015; Zheng et al., 2018; Alexander et al., 2021), LX 112 (Wanner and Vishwanathan, 2018; Kislinger et al., 2020), Glycid ether 100 (Ding et al., 2016; Wanner et al., 2016; Motta et al., 2019), and Poly/bed 812 (Takemura et al., 2013). Some of these choices may reflect known properties of resins and how they interact with tissues, such as the low viscosity of Spurr’s resin for rapid infiltration (Spurr, 1969) or stability under an electron beam (Kizilyaprak et al., 2015). Rarely, however, are the reasons for the choice of a particular resin made explicit in publications. We sought to explore whether a more quantitative approach would be informative when evaluating resins for ultrathin sectioning including monitoring the hardness of resin blocks as well as explicitly measuring the cutting forces during sectioning. Of primary importance was to characterize the repeatability of section thicknesses for ultrathin sections (<=50 nm) across hundreds of serial sections since this ultimately determines the quality of the sampling along the Z axis of volume EM datasets. Our ultimate goal was to define a test platform that can be used to identify an optimal embedding resin for ultrathin sectioning as well as to evaluate lot-to-lot variability and the suitability of future resins for volume EM.

The hardness of embedding resins has previously been reported and an optimal hardness for ultrathin sectioning was determined to be in a range of 13−18 HV (Vickers hardness) (Jésior, 1985). We extend this observation to quantify the time course of block hardening, block-to-block variability, factors leading to non-uniformity in the curing of blocks, and the impact of environmental variables on resin block hardness. Similarly, devices to quantify sectioning forces have been previously described (Helander, 1974; Ericson and Lindberg, 1997; Sun et al., 2017), but have not been used to systematically evaluate the cutting properties of commonly used embedding resins nor the impact of embedding heavily stained tissue samples on cutting forces. The quantification of cutting forces allows cutting repeatability (i.e., the difference in thickness between serial sections) and cutting uniformity (i.e., the absence of chatter) to be measured. Overall, we evaluated the sectioning of several common embedding resins at both 35 and 50 nm section thicknesses using resin block hardness and cutting forces as quantitative metrics. Because the definition of “good” sectioning has been rather qualitative to date, our results are aimed at electron microscopists seeking a more quantitative approach to selecting embedding resins.

## Materials and methods

### Epoxy sample preparation

**FIGURE 1:**
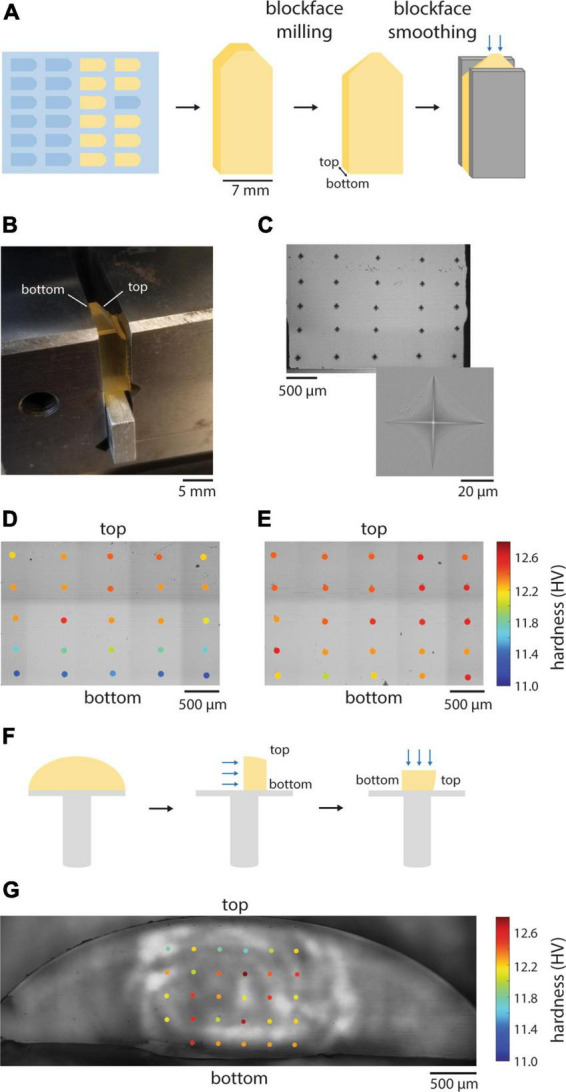
Hardness uniformity of resin blocks. **(A)** Illustration of the preparation of resin blocks for hardness testing. Blue arrows indicate the surface that was tested. **(B)** Resin block clamped in place for indentation hardness measurements. **(C)** A representative grid pattern for hardness mapping of a block face. Inset shows an individual diamond indentation from which hardness is calculated. **(D)** Hardness measurements from an EMbed 812 MH block cured in a silicon mold. **(E)** Hardness measurements from EMbed 812 MH resin that was first degassed prior to curing. **(F)** Illustration of the preparation of resin cross-sections that were cured on aluminum stubs for hardness testing. Blue arrows indicate the surface that was tested. **(G)** Hardness measurements from EMbed 812 MH resin that was first cured on a stub. Measurements were made from a cross-section of the dome of resin, perpendicular to the stub surface.

Samples for hardness testing were prepared by weighing the epoxy and hardener components (see Table 1) into a tared 125 ml glass Erlenmeyer flask or 50 ml centrifuge tube on either a precision (Kern) or analytical balance (Sartorius). After adding the components, the containers were placed in a 60°C oven (OV1, Biometra) for several minutes to reduce viscosity and mixed thoroughly by vortexing or vigorous shaking. After the mixtures became free flowing and streak-free, the respective accelerator was added by volume with a micropipette and mixed again. In preliminary studies, we used glass rods to mechanically mix the components, but also observed non-uniform hardness near the bottom of silicone embedding molds in these samples similar to the results reported in Figure 1D. An optional vacuum degassing step was included following the addition of the accelerator, in which the open container was placed in a desiccator (Mini Hot Vac, Ted Pella) and evacuated to approximately −70 kPa using a laboratory vacuum pump (KNF, Neuberger). The epoxy resin was degassed until no further bubble formation was observed, usually for 10−30 min. It was then pipetted onto aluminum pins (75638, EMS) that were previously scored with a razorblade for obtaining “stubs” or filled into flatbed silicone molds (70907, EMS) for blocks.

**TABLE 1:** Epoxy formulations used for this study.

	Epoxy					Hardener				Accelerator	
	***EMbed 812***	***LX 112***	***A***	***ERL 4221***	***DER 736***	***DDSA***	***NMA***	***NSA***	***B***	***BDMA***	***C***
*Density g/mL*	1.146	−	−	1.162	1.14	1.005	1.232	1.03	−	−	−
*Weight per epoxide (WPE)*	144	144	−	133.2	184.6	−	−	−	−	−	−
*EMbed 812 medium (M) epon*	4.20	−	−	−	−	2.95	1.67	−	−	0.300	−
*EMbed 812 medium hard (MH) epon*	5.96	−	−	−	−	3.02	2.71	−	−	0.240	−
*EMbed 812 -var I epon*	5.96	−	−	−	−	2.81	2.96	−	−	0.240	−
*EMbed 812 -var II epon*	5.96	−	−	−	−	2.61	3.20	−	−	0.240	−
*EMbed 812 -var III epon*	5.96	−	−	−		2.41	3.45	−	−	0.240	−
*EMbed 812 –hard (H) epon*	5.73	−	−	−	−	2.26	3.70	−	−	0.300	−
*EMbed 812 -very hard (VH) epon*	5.73	−	−	−	−	−	5.24	−	−	0.275	−
*Durcupan*	−	−	5.00	−	−	−	−	−	5.00	−	0.075
*LX 112*	−	6.26	−	−	−	−	3.88	1.88	−	0.240	−
*Spurr’s*	−	−	−	2.91	2.28	−	−	6.44	−	0.245	−

All epoxy and hardener quantities are reported in grams (except where noted), even if original protocols report ml. Accelerator quantity is reported in milliliters.

After curing in an oven (UN30plus, Memmert), samples were clamped in ultramicrotome chucks and first trimmed on a milling device (EM Trim2, Leica) before polishing on a microtome (UC7, Leica) with a diamond trimming knife (Trim20 or Trim90, Diatome) to a flat surface. For hardness tests, a rectangular blockface was trimmed at least 700 μm deep into a flatbed block with a 700 × 1400 μm^2^ block face. For cutting force tests, the blockface of a resin stub was trimmed to a hexagonal shape with dimensions 1.5 mm length, 0.9 mm width, a 110° leading edge and a 70° trailing edge.

The following resins were evaluated: EMbed 812 Kit (14121, EMS), Spurr’s Low Viscosity Kit (14300, EMS), Durcupan ACM Kit (44610, Sigma-Aldrich), and LX 112 Kit (21212, Ladd Research). We used NSA (19050, EMS) in our protocol for LX 112.

### Tissue sample preparation

Three *Danionella cerebrum* (∼3 mpf) were anaesthetized in 168 mg/L tricaine in Fish Ringer’s solution and brains were then excised and immersion fixed with 2% paraformaldehyde (15714, EMS) and 2% glutaraldehyde (GA) (16320, EMS) in 0.1 M sodium cacodylate buffer containing 5.5% sucrose at room temperature (RT) for 24 h. The brain samples measured approximately 0.8 × 1.5 × 2.0 mm^3^. All animal experiments were conducted in accordance with the animal welfare guidelines of the Max Planck Society and with animal experimentation approval granted by the Landesamt für Natur, Umwelt und Verbraucherschutz Nordrhein-Westfalen, Germany.

One sample was embedded in 12% gelatin (G2500, Sigma-Aldrich), fixed with 2% GA in 0.175 M sodium cacodylate buffer, and cut into a gelatin block prior to EM staining. Staining was performed either manually or in a tissue processor (EM TP, Leica). All samples were stained with reduced osmium [2% osmium tetroxide (19170, EMS), 3% K-ferrocyanide (60279, Sigma-Aldrich)] in 175 or 180 mM cacodylate buffer (20840, Sigma-Aldrich) for 4 h at 4°C, 1% TCH (21900, EMS) in water for 1 h at 45°C, and then 2% osmium tetroxide in water for 2 h at RT with intermediate washing steps (ROTO sample). Two samples were further processed by staining in 1% uranyl acetate (22400-2, EMS) in water for 12 h at 45°C and Walton’s lead aspartate (203580 and A7219, Sigma-Aldrich) for 6 h at 45°C, with washing steps between reagents (ROTO + UA + Pb samples). The gelatin block was black from the osmium staining with no visual access to the sample. One additional whole brain sample was gelatin-embedded as described above after staining resulting in a gelatin block that was transparent around the sample.

Tissue samples were dehydrated in a graded series of ethanol (70%/90%/100%/100%) (15055, EMS) followed by changes in propylene oxide (PO) (82320, Sigma-Aldrich) and infiltrated with EMbed 812 resin in the medium hard formulation (MH), first in a 50:50 dilution with PO, then with several changes in pure resin. Whole brains and brains in gelatin blocks were flat-embedded in silicone molds for sagittal cross-sectioning. Samples were cured in a 70°C oven for 48 h and, after exploratory sectioning, remounted on aluminum stubs with epoxy resin for cutting force tests.

### Hardness testing procedure

Samples were positioned in a hardness testing machine (Q10A+, Qness) and fixed with a vise (1111182, Röhm) with the blockface facing upwards. Using the manufacturer’s software, a 10x magnification overview image of the blockface was taken and areas selected for individual test points or test point grids. Vickers hardness testing then commenced in an automated approach, using a 136° diamond indenter applying 25 grams of force for 10 s. Images of the indentations were then automatically acquired using a 40x objective. Endpoints of the diagonals were manually annotated and the Vickers hardness (HV) was then automatically calculated within the Q10A+ software based on the machine calibration.

### Humidity tests

For humidity tests, samples were tested for hardness in a monitored lab environment or after incubation in a makeshift humidity chamber. This chamber was a small incubator (MyTemp Mini, Benchmark) lined with damp tissue paper or bags of desiccant for wetting and drying and a battery-powered hygrometer (30.5027.01, TFA). To alter the humidity, water was sprayed into the chamber or an amount of tissue paper exchanged with desiccant. After approximately 5 h per condition, samples were removed from the humidity chamber and immediately tested for hardness.

### Cutting force tests

A cutting force sensor was constructed as previously described (Ericson and Lindberg, 1997), with the minor alteration of using two push/pull piezoelectric sensors (PCB-209C11, PCB Piezotronics). The signals from the sensors were amplified (PCB-482C16), digitized (USB-6356, National Instruments) and recorded within Matlab (Mathworks). Continuous cutting tests were performed at either 35 or 50 nm section thickness with the UC7 microtome for a duration of 15 min for each resin tested. All cutting tests were performed at a speed of 1.2 mm/s, except for data in which sectioning speed was varied and sections were collected for 30 (at 0.6 mm/s) or 60 (at 0.3 mm/s) minutes. Videos were captured during the sectioning process to measure section compression. For each cut profile the sectional and transverse forces were calculated by subtracting or summing the sensor signals, respectively. The baseline signal before and after each cut was fit with a line and subtracted from each force profile. The slow discharge time constant of the force sensors was not corrected for the measurements of the mean cutting force, except for sections cut to assess the effect of sectioning speed in which a single exponential was fit to the mean force profiles during the portion of the cut corresponding to the parallel sides of the block. This single exponential was then deconvolved with the force profile in Matlab to account for the slow discharge time constant. All cutting force tests were performed with a 35° diamond knife set to a 6 degree clearance angle (Ultra 35 knife, Diatome), except for the tests that varied clearance angle. Note that the clearance angles we report do not include the built-in 4 degree clearance of the Ultra 35 knives.

## Results

We began by quantifying the hardness of our standard embedding protocol for EMbed 812, medium hard resin. We clamped resin blocks that had been trimmed and smoothed in a diamond indention hardness testing machine (Figures 1A, B) and performed hardness measurements in a grid pattern across a blockface (Figure 1C). The machine drops a calibrated weight on the resin block leading to a diamond-shaped indentation pattern, the shape of which is quantified and reported as Vickers hardness (HV) (Low and Shi, 1998). Our standard embedding protocol involved embedding tissue samples in 4 mm deep silicon molds and curing for 24−48 h at 70°C. We were surprised to measure a gradient in hardness values that varied from lower hardness in the deepest part of the block (bottom) to higher hardness toward the top (more superficial) regions of the blockface (Figure 1D). We hypothesized that either the oven heat was not equally distributed in the thermally insulating silicon mold or gas was trapped in the deeper portions of the embedding well. To test these possibilities, we degassed the resin in a weak vacuum before curing. The result was an improved homogeneity of hardness across block faces (Figure 1E). To explore whether a non-uniform distribution of heat contributed to the hardness gradient, we also cured non-degassed resin on aluminum stubs and measure hardness across a vertical cross-section relative to the stub surface (Figures 1F, G). We again observed an improvement in the distribution of hardness values, but to a lesser degree than the degassed resin. Overall, quantifying the distribution of hardness across block faces has changed the method by which we cure our specimens to achieve more uniformity and we now routinely degas and cure resins on thermally conductive stubs.

**FIGURE 2:**
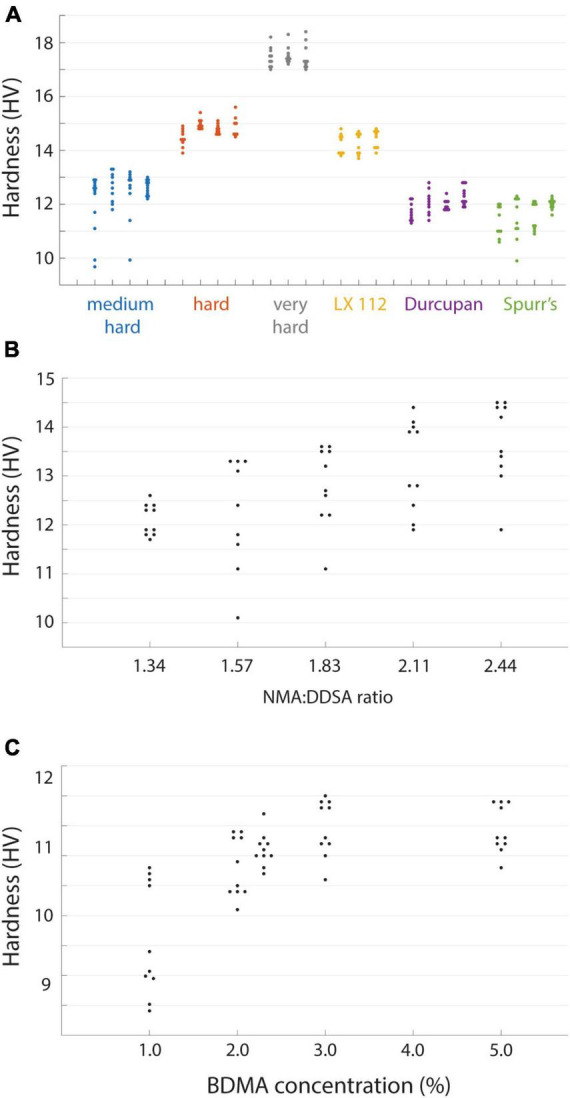
Hardness of different resin formulations. **(A)** Hardness values from independent blocks for each of the tested resins. Hardness (*n* = 8–20 points per block face) was measured for three or four blocks of each resin type. **(B)** The hardness of EMbed 812 measured for different ratios of hardeners NMA and DDSA. **(C)** Hardness of EMbed 812 MH cured with different concentrations of the accelerator (BDMA).

We next quantified the hardness of several commonly used embedding resins including EMbed 812 (with varying hardness formulations), Durcupan (Staeubli, 1963), Spurr’s (Spurr, 1969), and LX 112 (Stratton et al., 1982; Figure 2A). The different resins spanned a hardness range of ∼10−18 HV, but the hardness values measured between independent blocks were fairly repeatable. We selected EMbed 812, a replacement for Epon 812 (Luft, 1961), to explore varying the ratio of the hardeners typically used (DDSA and NMA). As previously reported (Luft, 1961), changing the ratio of these components allows one to titrate the hardness of the cured blocks (Figure 2B) in a range we term medium-hard (1.34 NMA:DDSA) to hard (2.44 NMA:DDSA). Omitting DDSA yielded the hardest block of all formulations we tested, the very-hard formulation (Figure 2A). We typically include the manufacturer recommended accelerator (BDMA) concentration of 2−3%. The hardness of EMbed 812 in this range and up to 5% concentration was similar, but notably yielded lower hardness below 2% (Figure 2C). We have not tested an alternative accelerator, DMP-30, that is sometimes used in commercial embedding kits.

**FIGURE 3:**
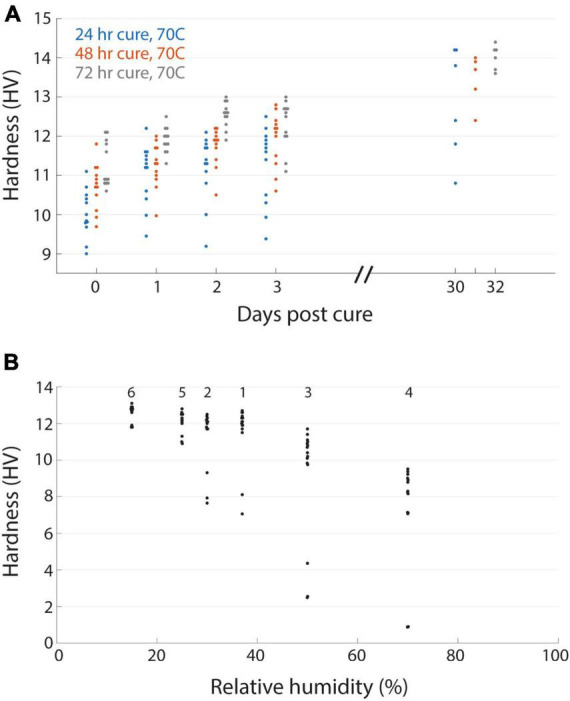
Time varying and environmental properties of resin hardness. **(A)** EMbed 812 blocks cured for 24 (blue), 48 (red), or 72 (grey) hours and then measured for hardness at different days post-cure. **(B)** Hardness of an EMbed 812 MH resin block measured at different relative humidities. Small numbers indicate the sequence in which measurements were performed.

Resin blocks can continue to harden at room temperature even after the initial high temperature curing process, either through ongoing polymerization or possibly the leaching of unreacted resin components. In some cases this is evident by a change in the color of blocks on the time course of months or years. To explore the evolution of hardness as a proxy for the curing state of a block, we tested different initial curing durations and then tracked the hardness of blocks over days out to a month (Figure 3A). We performed an initial cure at 70°C for 1, 2, or 3 days of the EMbed 812 MH formulation and observed an initial tendency for the longer duration oven cure to yield harder blocks. This tendency persisted for the first 3 days after the curing. When measured again approximately 1 month later, the same blocks had increased in hardness by approximately 2 HV, indicating a substantial post-cure hardening of the blocks. This time-dependency of the hardness complicated measurements, but we have tried to be as consistent as possible. For example, the measurements comparing individual blocks (Figure 2A) were performed 20−30 days after the initial cure and measurements relating hardness to cutting force were performed within 1 day of cutting force tests. In general, hardness measurements are useful to quantify the state of post-curing which can be important for serial sectioning experiments of tissue blocks that were recently prepared.

Resins are also known to absorb atmospheric moisture which can lead to poor embedding (Dellmann and Pearson, 1977). We had anecdotally observed that serial sectioning experiments were more error prone under higher humidity conditions. We quantified the relationship between relative humidity and hardness for a block that was placed in a humidifying or dehumidifying chamber and then immediately tested for hardness (Figure 3B). For relative humidity <40% the hardness was relatively stable, but already at 50% humidity we observed a reduction in hardness that was even lower at 70% humidity. The change in hardness was reversible as transitioning a block from high humidity (70%) to lower humidity (25%) recovered the hardness (Figure 3B). Because our laboratories are not centrally humidity controlled we now perform sectioning experiments in rooms in which industrial portable dehumidifiers are able to maintain a constant relative humidity of 30−45%, a range in which it is comfortable to work, to ensure stable block hardness.

### Cutting force measurement

**FIGURE 4:**
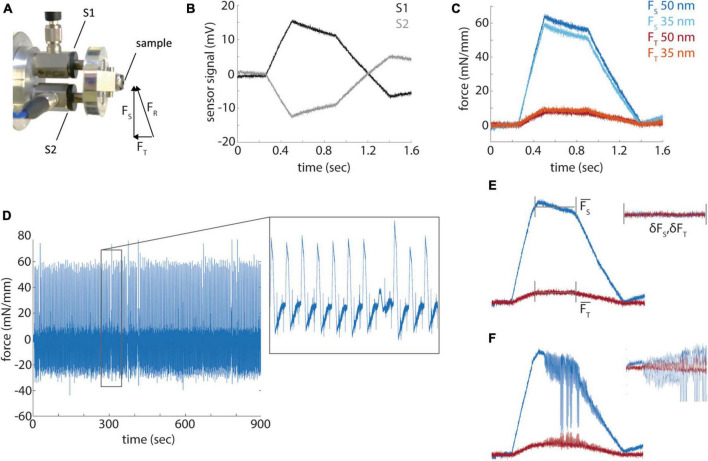
Force profile measurements. **(A)** A photograph of the force sensors (S1 and S2) mount and resultant, sectional and transverse forces measured by the sensors. **(B)** Raw signals from the force sensors during a section from a diamond shaped resin block. **(C)** Representative sectional (F_*S*_) and transverse (F_*T*_) forces for sections cut at 35 and 50 nm. **(D)** Sectional forces from a 15 min long sectioning session. Inset shows a magnified portion of the time series. **(E)** A diagram of the measurement of mean sectional and transverse forces and the standard deviation of the force during the cut. **(F)** A representative example of a cut with high frequency chatter.

**FIGURE 5:**
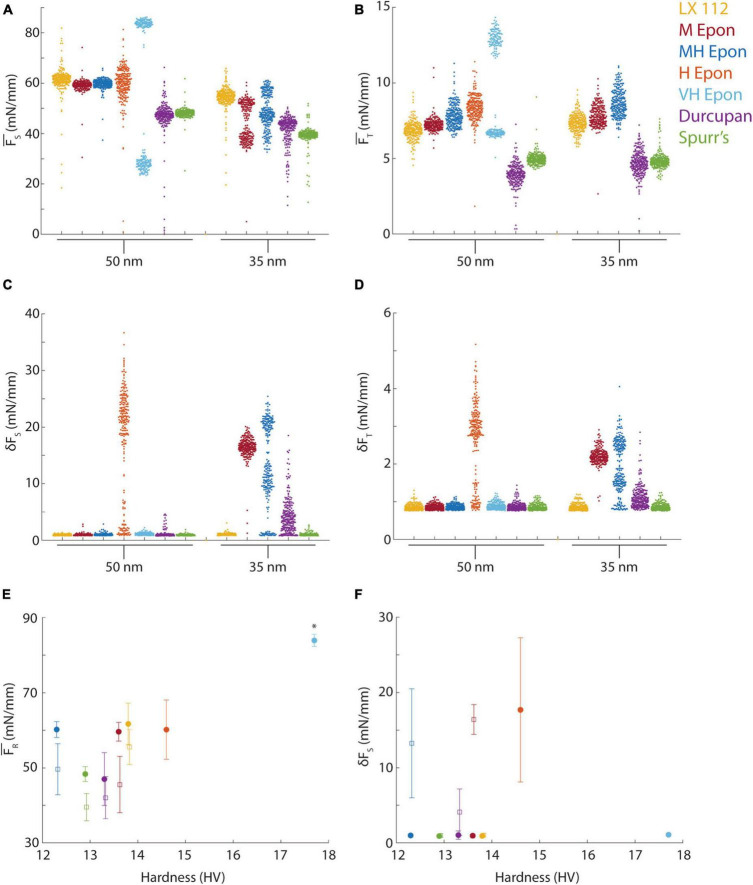
Force measurements from different resins. **(A)** Mean sectional force from different resins (color-coded) cut at 35 or 50 nm. **(B)** Mean transverse force from different resins (color-coded) cut at 35 or 50 nm nominal section thickness. **(C,D)** Standard deviation of sectional and transverse forces at 35 or 50 nm nominal section thickness. **(E)** Mean resultant forces plotted versus block hardness for 50 nm (filled circles) and 35 nm (open squares) sections. Asterisk indicates the forces for EMbed 812 VH resin were from ∼100 nm sections due to missed cuts. **(F)** Mean of the standard deviation of sectional force plotted versus block hardness for 50 nm (filled circles) and 35 nm (open squares) sections.

While the quantification of hardness allowed us to make several practical improvements to our standard block preparation and sectioning procedures, it is not a direct measurement of the quality of sectioning that can be obtained from different resins. Indeed, prior quantifications of the mechanical properties of embedding resins involved only qualitative evaluations of section quality (Acetarin et al., 1987). Previous work had described a method to instrument commercial ultramicrotomes with a force sensor attached to the sample arm to directly measure cutting forces (Ericson and Lindberg, 1997). We implemented this design with the minor change of using pre-stressed piezoelectric force sensors that are capable of sensing push and pull forces (Figure 4A). Because two sensors (S1 and S2) are symmetrically arranged around a sample along the cutting axis, the resultant force (F_*R*_) of a diamond knife into a sample can be decomposed into the sectional force (F_*s*_) along the blockface and transverse force (F_*T*_) into the block face. F_*S*_ is calculated as the difference of S1 and S2 and F_*T*_ as the sum of the two signals (Figures 4B, C). Sectioning forces are reported normalized to the width of a given block. The sectional force is sufficiently sensitive to distinguish relatively small differences in sectioning thickness, for example, 35 nm versus 50 nm sections (Figure 4C), whereas the transverse force is of lower amplitude and more difficult to distinguish section thickness (but see Figure 5B).

To quantify cutting quality, we define two terms: cutting repeatability (the variability in the mean sectional force among sections that correlates with section thickness variability) and cutting uniformity (the presence or absence of chatter). We performed 15 min long sectioning sessions at both 50 and 35 nm thickness that typically yielded ∼250 cuts at a sectioning speed of 1.2 mm/s (Figure 4D). The analysis of the cutting profiles revealed the variability of each cut, ranging from uniform cutting, missed cuts, alternating thickness cuts and chatter during a cut. We measured the mean sectional and transverse cutting forces during the parallel portion of the block face, excluding the leading and trailing ramps, for each block as well as the standard deviation of the high-pass filtered force profile (Figure 4E). Cutting repeatability is therefore the variability of the mean sectional force across cuts. The standard deviation of each cut (δF_*S*_, δF_*T*_) or cutting uniformity reveals changes in the force during the cut and shows higher variability when, for example, chatter develops during a cut (Figure 4F).

We proceeded to quantify the cutting forces at both 35 and 50 nm ultramicrotome feed settings from seven resin blocks, all trimmed to identical shapes, including LX 112, EMbed 812 medium (M), medium hard (MH), hard (H) and very hard (VH) formulations, Durcupan and Spurr’s resins. The mean sectional cutting forces ranged from ∼40 mN/mm to ∼70 mN/mm for the different resins (Figure 5A) and from ∼3 mN/mm to ∼10 mN/mm for the transverse force (Figure 5B). The EMbed 812 VH formulation skipped every other cut and therefore the bimodal distribution of forces likely reflects 100 nm rather than 50 nm sections. The missed cuts still yielded a measurable force (< 30 mN/mm) indicating the knife remained in contact with the block despite the lack of a section being cut. The variability in the mean cutting force (i.e., the spread of datapoints in Figures 5A, B) is a measure of cutting repeatability because it reveals variability in section thickness. For example, Durcupan had similar mean sectional forces as Spurr’s resin at 50 nm, but a worse cutting repeatability due to the larger variability in the distribution of cutting forces (Figure 5A). The transverse force was similarly variable regardless of resin formulation but the lowest mean transverse forces were measured for Spurr’s and Durcupan (Figure 5B).

Measuring the standard deviation of each cut (δF_*S*_, δF_*T*_) reveals cutting uniformity. For example, the EMbed 812 H formulation exhibited such excessive chatter during sectioning already at 50 nm that we did not test it at 35 nm (Figures 5C, D). In several cases, such as Durcupan and EMbed 812 M and MH, the cutting uniformity was worse at 35 nm compared to 50 nm. Similarly, the EMbed 812 VH formulation that sectioned at ∼100 nm instead of 50 nm due to skipped cuts resulted in better cutting uniformity, consistent with less chatter developing in the thicker sections. Among the tested resins, only Spurr’s and LX 112 yielded reasonably good cutting repeatability and cutting uniformity at both 35 and 50 nm section thicknesses.

Because we had measured the blockface hardness profiles of each block before performing cutting force tests, we explored whether the mean hardness of a block was a predictor of its cutting properties by plotting the resultant (total) force versus hardness (Figure 5E). We found that resins with hardness near ∼13 HV resulted in lower mean cutting forces, but hardness did not obviously correlate with cutting repeatability across the different resin formulations (Figure 5E, see standard deviation around the mean). Overall, Spurr’s resin exhibited the lowest cutting forces and best cutting repeatability at both 35 and 50 nm, but we have not tested the effect of preparing slightly softer or harder Spurr’s blocks. The cutting uniformity (δF_*T*_) was also not obviously correlated with block hardness (Figure 5F). For example, Durcupan, EMbed 812 M and MH all exhibited chatter at 35 nm despite spanning a range of hardness from 12.3 to 13.8 HV.

### Section compression

**FIGURE 6:**
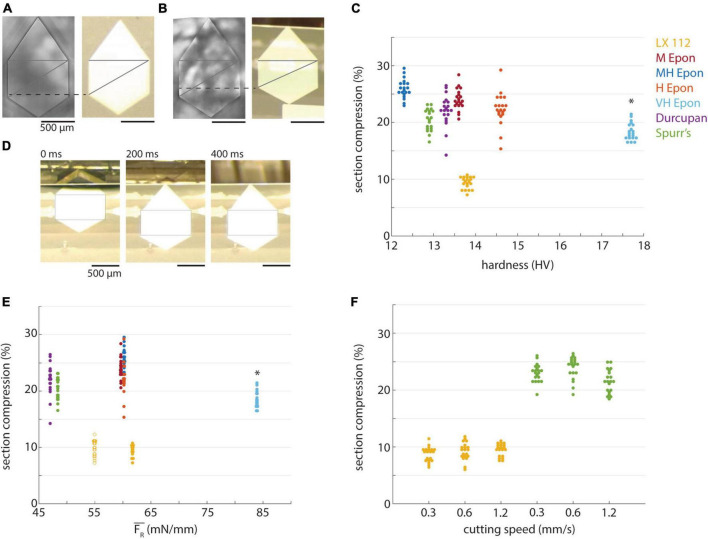
Compression of different resins. **(A)** A block of LX 112 resin (left) and representative 50 nm section (right). Lines indicate method used to measure compression along cutting direction. **(B)** A block of EMbed 812 MH resin (left) and representative 50 nm section (right). **(C)** Percentage of section compression relative to the length of block faces plotted versus hardness for different resins (color-coded, 50 nm sections). Asterisk indicates the compression percentages for EMbed 812 VH resin were from ∼100 nm sections due to missed cuts. **(D)** Decompression of a 50 nm LX 112 section on the water surface at different time points. Rectangular box is equally sized in the three images. **(E)** Percentage of section compression plotted versus mean resultant force (color-coded). Open circles represent compression of 35 nm LX 112 sections, closed circles are 50 nm sections. Asterisk indicates the compression percentages and forces for EMbed 812 VH resin were from ∼100 nm sections due to missed cuts. **(F)** Percentage of section compression plotted versus cutting speed (color-coded, 50 nm sections).

Sections are frequently compressed along the cutting direction during sectioning (Jésior, 1985, 1986). Such compression can be undesirable because it distorts the morphology of structures within tissue samples, although sections can in principle be decompressed on the surface of a water boat with heat or chloroform vapor (Sotelo, 1957; Peachey, 1958). We quantified the compression of 50 nm sections for each of the resins relative to dimensions of the block face (Figures 6A, B) and found that sections from most resins compressed by factor of ∼15−25% (Figure 6C). There was a slight downward trend in section compression for the harder EMbed 812 resin blocks, consistent with a previous report that compression is inversely correlated with resin hardness (Jésior, 1985), but this is likely influenced by the thicker EMbed 812 VH sections. A notable exception was the LX 112 resin which compressed substantially less than all other resins (∼10%). The decompression of LX 112 sections was noticeably visible on the water boat surface within the first few 100 ms after each cut (Figure 6D). We did not observe an obvious correlation between the magnitude of compression and block hardness (Figure 6C) or the resultant cutting force (Figure 6E). The ∼10% section compression for 50 nm LX 112 sections was also observed for 35 nm sections (Figure 6E, open circles). For Spurr’s and LX 112 resins, we tested whether section compression was dependent on cutting speed and found no correlation (Figure 6F) as was previously reported (Jésior, 1986).

### Effect of cutting parameters on cutting repeatability and uniformity

**FIGURE 7:**
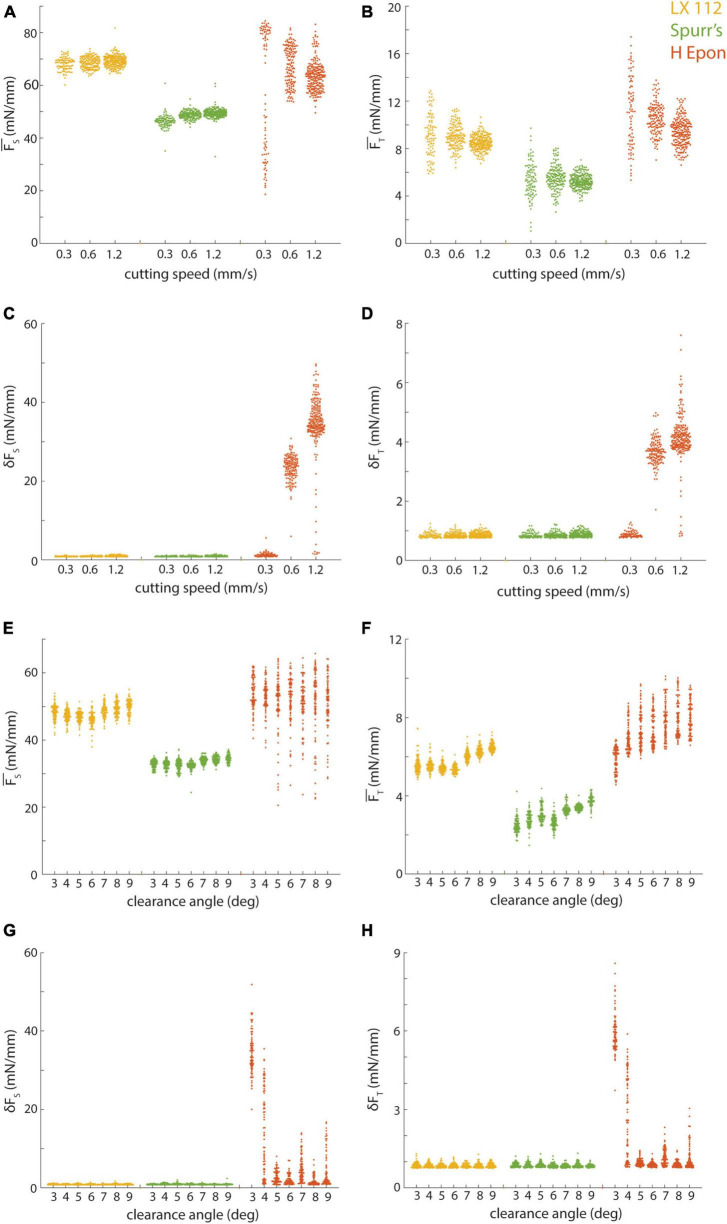
Effect of cutting parameters on cutting repeatability and uniformity. **(A,B)** Mean sectional and transverse forces from different resins (color-coded) cut at 50 nm and 6 degree clearance angle plotted versus cutting speed. **(C,D)** Standard deviation of sectional and transverse forces from different resins (color-coded) cut at 50 nm and 6 degree clearance angle plotted versus cutting speed. **(E,F)** Mean sectional and transverse forces from different resins (color-coded) cut at 35 nm and 1.2 mm/s plotted versus knife clearance angle. **(G,H)** Standard deviation of sectional and transverse forces from different resins (color-coded) cut at 35 nm and 1.2 mm/s plotted versus knife clearance angle. Note that reported clearance angles do not include the built-in 4 degree clearance angle of the knives we used.

We opted to hold several key cutting parameters constant for the preceding cutting force tests (Figure 5) including cutting speed (1.2 mm/s) and clearance angle (6°). However, it is possible that improved cutting repeatability and uniformity could be achieved by further optimizing these parameters for each resin. In particular, while we have obtained excellent results cutting at 1.0−1.2 mm/s with our current collection methodology (Fulton et al., 2023), this speed is substantially faster than that used for most serial section volume EM studies. We therefore cut additional 50 nm series for LX 112, Spurr’s, and EMbed 812 H resins at 0.3 and 0.6 mm/s (Figures 7A–D). For LX 112 and Spurr’s, we observed comparable results in terms of cutting repeatability and uniformity across the different cutting speeds. For EMbed 812 H, we observed an improvement in cutting uniformity (reduced chatter) as the cutting speed was lowered (Figures 7C, D), but a reduction in cutting repeatability at the slowest cutting speed (Figures 7A, B).

We also tested cutting 35 nm sections with a range of clearance angles (3−9°, cut at 1.2 mm/s) for these three resins and found that LX 112 and Spurr’s cut with a similar repeatability and uniformity regardless of clearance angle (Figures 7E–H). The repeatability of EMbed 812 H was similar across the range of clearance angles (Figures 7E, F), but the uniformity degraded at the shallowest cutting clearance angles tested (Figures 7G, H). Overall, while it is clear that the cutting parameter space for each resin should ideally be individually optimized, Spurr’s and LX 112 were relatively robust in terms of cutting repeatability and uniformity to many combinations of cutting speeds and clearance angles.

### Differential tissue/resin hardness

**FIGURE 8:**
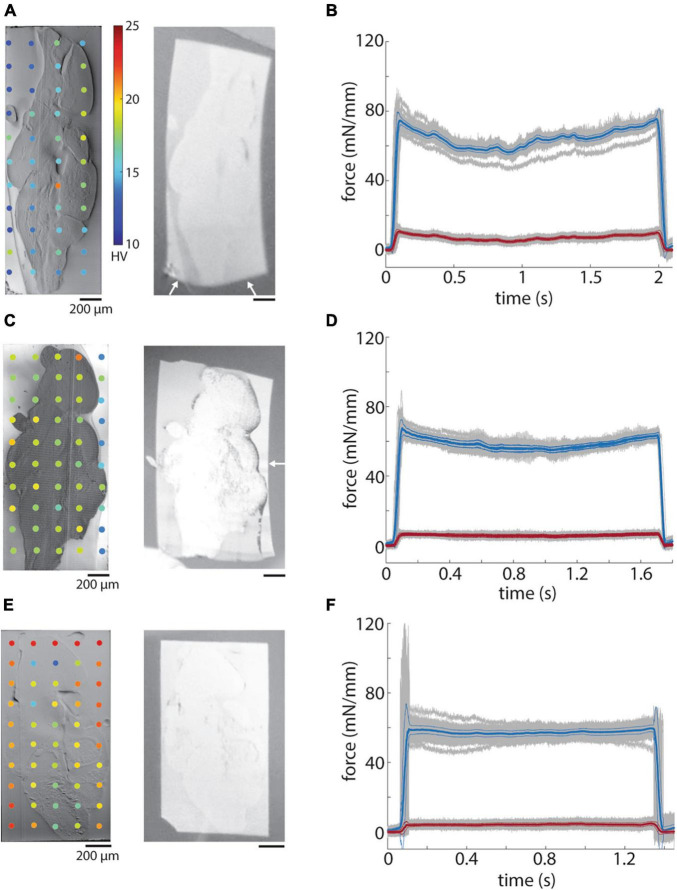
Hardness of embedded tissue samples. **(A)** Blockface image of a ROTO stained brain embedded in EMbed 812 MH resin (left) and a 50 nm section (right). White arrows indicate distortion of the section. **(B)** Mean sectional (blue) and transverse (red) forces for 50 nm sections from the block in panel A. **(C)** Blockface image of a ROTO + UA + Pb stained brain surrounded first with 12% gelatin and then embedded in EMbed 812 medium hard resin (left) and a 50 nm section (right). White arrow indicates distortion of the portion of the section not containing gelatin. **(D)** Mean sectional (blue) and transverse (red) forces for 50 nm sections from the block in panel C. **(E)** Blockface image of a ROTO + UA + Pb stained brain surrounded first with 12% gelatin stained with ROTO and then embedded in EMbed 812 medium hard resin (left) and a 50 nm section (right). **(F)** Mean sectional (blue) and transverse (red) forces for 50 nm sections from the block in panel E.

Finally, we measured the hardness of a tissue sample stained with either ROTO (see Methods, Figure 8A) or ROTO + UA + Pb (see Methods, Figures 8C, E) relative to the surrounding resin (EMbed 812 MH). The hardness of embedded stained tissue was much higher (>15 HV) than most of the tested resins and was higher for more intensely stained samples (Figures 8C, E versus 8A). For irregularly shaped tissue samples that do not fully span the block face this leads to a strong inhomogeneity in the block face hardness. One correlate of this inhomogeneity is a distortion of the surrounding resin relative to the stained tissue in ultrathin sections (Figure 8A, right panel, white arrows). We also often observed wrinkles form at the transition between the tissue and resin.

We attempted to equilibrate the hardness difference between heavily stained tissue and resin by surrounding samples with 12% gelatin prior to the tissue dehydration and embedding in EMbed 812 MH. Gelatin was chosen due to its simplicity to work with and optical transparency. Gelatin alone raised the hardness to a level roughly equal to that of ROTO + UA + Pb stained samples (Figure 8C). We also explored staining the gelatin with ROTO and found a further increase in hardness to a level exceeding that of the stained tissue sample (Figure 8E). Sections from both the unstained and stained gelatin samples exhibited less distortion of sections on the water surface (Figures 8C, E, right panels). Finally, we performed force measurements of the samples and found a larger deviation of the sectional force for the sample without any surrounding gelatin (Figure 8B) compare to the samples with gelatin (Figures 8D, F). Overall, the pre-embedding of irregularly shaped samples with gelatin helps create a more uniform hardness across block faces, less distorted sections, and a lower variability in the cutting force.

## Discussion

We sought to define quantitative metrics to evaluate a range of currently available embedding resins. The use of indentation-based hardness measurements allowed us to achieve more uniform block faces (Figure 1), based on the assumption that uniformly hard blocks are desirable, as well as to monitor the degree of post-cure hardening of blocks and the impact of humidity (Figure 3). Measuring cutting forces during sectioning allowed us to quantify cutting repeatability and uniformity, two key metrics to generate high-quality volume EM datasets (Figures 4, 5). Spurr’s resin, in particular, performed well for ultrathin sectioning down to 35 nm relative to the other tested resins at least for the cutting parameters we selected. It has been previously speculated that minimizing sectioning forces yields the best quality sections (Ericson and Lindberg, 1997). In line with this, Spurr’s exhibited among the lowest total sectioning forces at 35 nm and best cutting repeatability, but LX 112 also cut reasonably well at 35 nm despite higher sectioning forces. Further measurements are needed that involve optimizing the cutting parameters for each resin to fully explore whether minimizing cutting forces in general leads to the best cutting repeatability and uniformity (Figure 7).

Overall, we observed a tendency of block hardness in the range of 13−14 HV to yield lower sectioning forces compared to softer or harder blocks. However, the mean hardness of block faces did not obviously correlate with cutting quality across the different resins. The degree of section compression was also not obviously correlated with hardness nor the magnitude of cutting forces (Figure 6). The mechanisms of section compression have been previously studied (Jésior, 1985, 1986) and, for example, the dependency of compression on section thickness and clearance angle suggests that hardness alone would be insufficient to predict compression. LX 112 sections visibly decompressed on the water surface after each section was cut, a property we did not observe for the other resins. This decompression may reflect the elastic relaxation of the LX 112 polymer or potentially an interaction between the polymer and water. We note that LX 112 was prepared with a different hardener than the EMbed 812 resins (NSA versus DDSA) and we speculate this may be related to the difference in section compression, but further investigation is needed.

We observed that irregularly shaped tissue samples embedded in resin can lead to distortions in ultrathin sections, particularly in the surrounding regions of bare resin (Figure 8). Potential residual stresses in blocks that result from differences in the coefficients of thermal expansion of the resin and tissue could explain such an effect (Hodges et al., 1989; Serbena and Zanotto, 2012; Luo et al., 2020). A modified curing schedule has been suggested to alleviate such stresses (Lu et al., 2023) and could help differentiate whether the hardness gradient between tissue and resin we measured is related to these residual stresses. The gelatin block in which we surrounded tissue prior to dehydration and embedding likely acts as an interphase, similar to a wide variety of polymer composites, to alter the viscoelastic properties of the block and smooth the transition between the tissue and neighboring resin (Sienkiewicz et al., 2022; Gkaliou et al., 2023). The gelatin may also help prevent the development of residual stresses during curing. The epoxy resin likely covalently binds to gelatin similar to molecules in the tissue itself which could smooth the transition between tissue and the surrounding resin (Sung et al., 1996).

There are several future extensions we anticipate for the use of hardness and cutting force measurements as quantitative metrics to evaluate resins. The resins we chose to characterize span a range of chemical structures including aliphatic (LX 112), cycloaliphatic (Spurr’s), and aromatic (Durcupan ACM) monomers as well as mixtures reported to contain both aliphatic and aromatic monomers (EMbed 812) (Glauert and Lewis, 1998). There are many resin formulations that we have not yet evaluated, including, for example, additional Araldites (beyond Durcupan ACM) (Glauert et al., 1956) and Quetol (Kushida, 1974), a water miscible resin. We also have not systemically measured post-cure hardening beyond a month, nor the impact of humidity on resins other than EMbed 812 MH. We emphasize that there are also numerous additional physical properties of resin blocks that could be quantified and ultimately correlated with cutting quality such as the glass transition temperature, fracture toughness, elastic modulus and tensile strength (Acetarin et al., 1987; Sun et al., 2017). For hardness measurements, we note that epoxy samples do not behave in a rigid-plastic manner, but also exhibit noticeable elastic properties. As a result, elastic recovery of indentations may lead to greater variability in the results from hardness testing. Therefore, we chose a load and application time that yielded the most robust hardness values for our samples. Finally, because our focus was on ultrathin sectioning, we have not yet assessed other important properties such as stability under exposure to an electron beam for the resins we tested (Kizilyaprak et al., 2015). Overall, hardness testing and cutting force measurements are relatively simple to implement in the laboratory and provide a more principled method to optimize sample embedding and choose an embedding resin compared to trial-and-error approaches.

## Data availability statement

The raw data supporting the conclusions of this article will be made available by the authors, without undue reservation.

## Ethics statement

The animal study was approved by the Landesamt für Natur, Umwelt und Verbraucherschutz Nordrhein-Westfalen, Germany. The study was conducted in accordance with the local legislation and institutional requirements.

## Author contributions

LT: Data curation, Formal analysis, Investigation, Methodology, Visualization, Writing – review & editing. KB: Data curation, Formal analysis, Conceptualization, Funding acquisition, Investigation, Methodology, Supervision, Writing – original draft, Writing – review & editing.
